# Focus on Chronic Exposure for Deriving Drinking Water Guidance Underestimates Potential Risk to Infants

**DOI:** 10.3390/ijerph15030512

**Published:** 2018-03-14

**Authors:** Helen Goeden

**Affiliations:** Minnesota Department of Health, St. Paul, MN 55164-0975, USA; helen.goeden@state.mn.us; Tel.: +01-651-201-4904

**Keywords:** drinking water guidance, infant exposure, chemical risk assessment, duration extrapolation

## Abstract

In 2007, the Minnesota Department of Health (MDH) developed new risk assessment methods for deriving human health-based water guidance (HBG) that incorporated the assessment of multiple exposure durations and life stages. The methodology is based on US Environmental Protection Agency recommendations for protecting children’s health (US EPA 2002). Over the last 10 years, the MDH has derived multiple duration (e.g., short-term, subchronic, and chronic) water guidance for over 60 chemicals. This effort involved derivation of multiple duration reference doses (RfDs) and selection of corresponding water intake rates (e.g., infant, child, and lifetime). As expected, RfDs typically decreased with increasing exposure duration. However, the corresponding HBG frequently did not decrease with increasing duration. For more than half of the chemicals, the shorter duration HBG was lower than chronic HBG value. Conventional wisdom has been that chronic-based values will be the most conservative and will therefore be protective of less than chronic exposures. However, the MDH’s experience highlights the importance of evaluating short-term exposures. For many chemicals, elevated intake rates early in life, coupled with short-term RfDs, resulted in the lowest HBG. Drinking water criteria based on chronic assessments may not be protective of short-term exposures in highly exposed populations such as formula-fed infants.

## 1. Introduction

Guidance values produced by human health risk assessments most often use animal experiments of different durations and key endpoints of consideration to derive a quantitative guidance value for the matrix of concern (water, air, soil, etc.). Chronic duration studies have been the preferred source of toxicity information, as these longer duration studies provide ample time for toxic effects to manifest from the exposure protocol. In recent years, a realization has been building that elevated sensitivity or exposures during the developmental time period may provide data that drive a risk assessment to a lower guidance value compared to use of longer duration chronic studies. In 1993, the National Academy of Science (NAS) [1] recommended changes to regulatory practice in order to ensure proper characterization of risks to infants and children. The specific recommendations included consideration of greater physiological sensitivity of infants and children, using exposure estimates representative of infants and children, and accounting for nondietary as well as dietary sources of exposure. The NAS report prompted passage of the Food Quality and Protection Act and amendments to the Safe Drinking Water Act related to children’s health protection. The US Environmental Protection Agency (US EPA) subsequently established the reference dose/reference concentration (RfD/RfC) Technical Panel early in 1999 to evaluate the RfD/RfC derivation process with respect to how well children and other potentially sensitive subpopulations are protected. As a result of this review, the RfD/RfC Technical Panel recommended derivation of RfD/RfCs for multiple exposure durations as well as enhanced or additional testing protocols to improve children’s health protection as part of the noncancer assessment process [2]. 

In 2007, the Minnesota Department of Health (MDH) began implementing exposure duration-specific methods that were adopted into state rule in 2009 [3]. For the derivation of noncancer health-based guidance, the MDH relied heavily upon the RfD/RfC Technical Panel recommendations [2] and additional duration- and life-stage specific US EPA guidance [4,5]. In addition, changes to MDH practice were mandated in Minnesota’s 2001 Health Standards Statute, which required that safe drinking water standards include “a reasonable margin of safety to adequately protect the health of infants, children, and adults …” [6]. This statute was based on concerns regarding children’s susceptibility to environmental contaminants. In response, the MDH’s approach to multiple-duration guidance included consideration of “windows of sensitivity” to toxicants as well as periods of high exposures. 

The MDH derives human health-based guidance (HBG) to evaluate potential health risks of environmental exposures. The focus of this manuscript are HBGs developed in response to concerns over contaminated water. MDH guidance is used by state programs as regulatory tools for groundwater protection, the prevention of exposure, remediation of contaminated sites, and to provide context for risk management. The MDH also uses HBG to advise households about chemical contamination in private wells. An HBG represents a water concentration (expressed as micrograms of chemical per liter of water, μg/L) that can be consumed with no appreciable risk to human health. HBGs are based on chemical toxicity (the dose that results in adverse effects), the duration of exposure (the time period of exposure that results in adverse effects), and the amount of water individuals drink during the exposure period.

Until 2007, the MDH used methods for deriving HBG that were similar to those used by the US EPA Office of Water for calculating chronic (lifetime) health advisories [7]. The US EPA methods for assessing noncancer health effects from lifetime exposure generally combine an adult water intake rate (2 L per day for a person weighing 70 kg, 2 L/70 kg b.w./day) with a chronic reference dose (RfD) and a relative source contribution factor (RSC) of 0.2. The RSC factor represents the maximum amount of the RfD “apportioned” to drinking water [8]. US EPA also derives 1- and 10-day health advisories using a child water intake rate (1 L per day for a child weighing 10 kg, 1 L/10 kg b.w./day) and no RSC (allowing 100% of the RfD to be from consumption of water). This approach typically results in short-term health advisory values that are greater than longer-term lifetime health advisory values. In contrast, the MDH’s implementation of exposure duration-specific methods has resulted in HBGs that have been more frequently driven by less-than-chronic exposure scenarios.

As of December 2017, the MDH has utilized the multiduration methodology discussed above to derive HBG for 85 chemicals or chemical groups. A wide range of chemicals have been evaluated, including, solvents, pesticides, consumer product/personal care-related chemicals, pharmaceuticals, and perfluorinated compounds. This publication is a retrospective evaluation of the MDH’s decade long experience of incorporating multiple duration evaluations into their process for deriving HBGs for water. The specific objectives of the evaluation are to: (1) evaluate the significance of incorporating multiple duration assessment methodology for deriving HBG intended for the general population (rather than the traditional single chronic duration) and (2) determine the overall utility of conducting multiple duration assessments for public health protection. 

## 2. Materials and Methods

To derive HBG, the MDH followed the standard US EPA four-step risk assessment process: hazard identification; dose–response/toxicity evaluation; exposure assessment; and risk characterization [9]. Hazard identification is a determination of the type of adverse effects posed by a chemical or substance. Dose–response or toxicity evaluation describes the quantitative relationship between the amount of exposure (i.e., dose) to a substance and the extent of toxic injury or disease. Exposure assessment typically includes the magnitude, frequency, duration, and route of exposure. Finally, when applied to developing standards or guidance, risk characterization integrates all three previously mentioned steps to derive a level of exposure that is unlikely to pose a health risk to the population of interest.

### 2.1. Hazard Assessment and Dose–Response

The chemicals chosen for review were identified by state agencies and the public as chemicals known or anticipated to be groundwater or surface water contaminants in Minnesota. For each chemical, government reports and peer-reviewed literature were searched for toxicity study information that encompassed various life stages and durations. The MDH adopted the duration definitions as recommended by the US EPA [2], as shown in Table 1. 

For each chemical assessment and each toxicity bioassay, the MDH evaluated information regarding the specific periods of time (e.g., life stage most susceptible to a toxic effect) and durations of exposure necessary to elicit an adverse effect. The MDH found that data were often not available for all dose–response time points or all life stages of potential interest, such as fetal or neonatal life stages. In addition, the time-point or exposure duration associated with effects was often not clear, since many parameters (e.g., histopathology) were assessed only at study termination. Studies that did include exposure during development (e.g., gestation, birth, and nursing) typically did not report the dose to the fetus or neonate. In these cases, the MDH used the maternal dose as a surrogate. 

Studies that did not include developmental exposure were useful for assessing shorter-duration dose–response for non-developmental health endpoints. Unless data to the contrary were available, these non-developmental endpoints (e.g., hepatotoxicity) were assumed to be relevant to any age group, including infants and children, when matched to the appropriate exposure duration. 

#### Reference Dose Derivation and Selection

Using the systematic approach recommended by the US EPA [2], the MDH derived an RfD for each duration for which sufficient toxicity data were available. An appropriate point of departure (e.g., no observable adverse effect level (NOAEL), lower confidence limit of a benchmark dose (BMDL), or lowest observable adverse effect level (LOAEL)) was identified and adjusted by applicable human equivalent dose conversion [10], uncertainty, and variability factors [3].

Since the RfD for each duration should be protective of all health endpoints that could occur within the given duration of exposure [2,3], the MDH considered and compared the short-term, subchronic, and chronic duration-based RfDs for a chemical prior to final RfD determination. Standard short-term, subchronic, and chronic toxicity bioassays typically do not encompass all life stages or may not assess all sensitive endpoints. In cases where the shorter-duration RfD was lower (more protective) than a longer-duration RfD due to life stage sensitivity or health effect assessed in the shorter duration study but not assessed in the longer-duration studies, the shorter-duration RfD was used as the longer-duration RfD as well. For example, a short-term RfD, based on developmental effects, may be lower than the chronic RfD based on evaluation of adult animals only. Since assessment of early life stages was not included in the chronic studies, the final chronic RfD would be set at the lower short-term RfD to ensure that short-term exposures that occur within a chronic durations are adequately protected. Shorter duration RfDs that were lower because of differences in study dose selection were not used as RfDs for longer durations. For example, wider dose spacing in the subchronic study could result in a lower subchronic RfD because the subchronic NOAEL was lower than the chronic NOAEL. The differences in the RfDs is due to the different dose levels selected alone and not due to the lack of life stage or endpoint assessment. This approach is consistent with US EPA recommendations [2] and recent US EPA practice [11]. Specific case where these decisions were made are described in Section 3.1.

The duration specific RfD and its basis for each chemical is available on the MDH’s website (http://www.health.state.mn.us/divs/eh/risk/guidance/gw/table.html) in the form of chemical-specific toxicity summaries. Links to the chemical specific summaries are also provided in Table S1 of the Supplemental Information. 

### 2.2. Exposure Assessment—Drinking Water Intake Rates and the RSC

Estimation of human doses from drinking water exposures requires a drinking water intake rate for assessing exposure in the context of the RfD. An HBG is derived by incorporating a drinking water intake rate, which is the quantity of water consumed per kilogram of body weight per day (L/kg per day). In 2007, the MDH used the US EPA’s report, “Estimated Per Capita Water Ingestion and Body Weight in the United States—An Update” [12] as a source of intake rates for the general population and subpopulations (e.g., children, pregnant women). As recommended by the US EPA Science Advisory Board (SAB) [13], the MDH used estimates of intake based on data for individuals who drank at least some tap water (i.e., consumer-only). The US EPA has since updated the Exposure Factors Handbook [14], which now serves as the source of water intake rates for the MDH’s HBG (see Table 2).

Infancy and early childhood are clearly periods during which exposure to water is the greatest, as demonstrated by the much larger water intake rates on a per body weight (L/kg per day) basis. Intake rates drop sharply with age, approximating adult rates on a per body weight basis by 6 to 11 years of age. The MDH selected the 95th percentile intake rates for HBG derivation. This ensured that the HBG was protective of individuals who consume most of their water from a single source, such as a private well.

For deriving short-term duration HBG, the MDH selected the 1 to <3 months old infant intake rate of 0.285 L/kg per day. For longer-durations, the MDH calculated time-weighted averages (TWA) starting at birth. The resulting subchronic (birth to approximately 8 years) and chronic (birth to approximately 70 years) duration TWA intake rates were 0.070 and 0.044 L/kg per day, respectively. These intake rates represent default values. Other exposure values were used if sufficient chemical-specific information indicated that a different duration or intake rate was more appropriate. For example, if a developmental effect was identified as the most sensitive effect and the susceptible period was limited to *in utero* development for the health effect of concern (e.g., cardiac malformations), the intake rate for pregnant women was used to calculate the HBG value.

Another aspect of exposure is the relative contribution that ingestion of water adds to the total exposure to a chemical. Water intake may constitute only one of several exposure pathways. A relative source contribution (RSC) factor is used to account for non-water ingestion related exposures (e.g., inhalation of volatilized chemicals, dermal absorption) as well as exposures via other media (e.g., consumer products, food, air, and soil) to ensure that the cumulative exposure does not exceed the RfD.

The use of an RSC is mandated by Minnesota state statute [15] and is based on the US EPA’s historic use of the RSC to derive drinking water health advisories and ambient water quality criteria. US EPA guidance for the latter includes an exposure decision tree for selecting RSC values [8]. The decision tree consists of a series of decision points at which the availability and quality of chemical and exposure data are evaluated. In general, a lack of statistically significant exposure data will steer the process towards a lower, more protective, RSC value. Higher RSC values may result if situation-specific data indicate that alternate exposures may not be as significant as water ingestion. US EPA [8] recommends that RSC values stay within the range of 0.2 to 0.8; the lower end of the range protects against other routes of exposure when uncertainty is high, and the upper end of the range allows for unknown exposures when uncertainty is low. Because the HBG are derived for exposure to the general public rather than a site-specific situation the application of the decision tree results in an RSC of 0.2 for most chemicals, which means that ingestion of water accounts for 20 percent of the RfD, with the remaining 80 percent coming from other pathways or sources of exposure. 

A default RSC of 0.2 was used for the multiple duration HBG developed for most of the 85 chemicals studied, with some exceptions. The narrower range of environments encountered by an infant during the first few months of life justified the use of an RSC of 0.5 for short-term (infant) exposures to most chemicals found in drinking water. The MDH used an RSC of 0.2 for all durations (including short-term based on infants) for chemicals that were classified as highly volatile, because inhalation is likely to be a significant exposure pathway for all durations. For pharmaceuticals available only through prescription, the MDH used an RSC of 0.8 for all durations because exposure from non-water sources, apart from prescription use, is unlikely. The MDH’s derivation of the default RSC values using US EPA’s decision tree process is documented in Appendix K of the MDH’s technical support document [3].

### 2.3. Risk Characterization

Understanding the relationship between timing and duration of exposure to a chemical and the subsequent adverse effects was essential in completing the characterization of risk. Calculation of each HBG combines a duration-specific RfD with the corresponding intake rate and a relative source contribution factor (RSC)

(1) $${\mathrm{nHBG}}_{\mathrm{duration}}=\;\frac{{\mathrm{RfD}}_{\mathrm{duration}}\;\times \;\mathrm{RSC}\;\times \;1000\;\mu g/\mathrm{mg}}{{\mathrm{IR}}_{\mathrm{duration}}}$$

where:

nHBG_duration_ = the noncancer health-based guidance value, for a given duration (see Table 1), expressed in units of micrograms of chemical per liter of water (µg/L).
RfD_duration_ = the reference dose, for a given duration (see Table 1), expressed in units of milligrams of the chemical per kilogram of body weight per day (mg/kg per day).
RSC = the relative source contribution factor, as described above.
IR_duration_ = the intake rate of water ingested for a given duration, as described above.

Departures from the HBG algorithm and parameter values discussed above were made if sufficient, appropriate chemical-specific information was available. The MDH rounded the HBG value to one significant figure, which was the least precise parameter used in the calculation.

#### Final Selection of Health-Based Guidance (HBG)

Similar to the decision process used for selection of RfDs, the MDH compared the longer-duration HBG with the corresponding shorter-duration HBG prior to determining the final health-protective HBG value. If the shorter-duration HBG value was lower than a longer-duration value, the shorter-duration HBG value was selected as guidance for the longer-duration exposure. This ensures that shorter-duration exposures that could occur within the longer-duration exposure period are protected.

A detailed description of the MDH’s methodology for deriving drinking water HBG was published in a technical support document [3]. The MDH’s application of this methodology, in the form of chemical-specific toxicological summaries, is publically available on the MDH website (http://www.health.state.mn.us/divs/eh/risk/guidance/gw/table.html).

## 3. Results and Discussion

Of the 85 chemical assessments conducted between 2007 and 2017, the MDH was able to derive short-term, subchronic and chronic RfDs and HBG in 63 (~74%) of the assessments. Subchronic and chronic (but not short-term RfDs) and HBG were derived for an additional 15 chemicals, for a total of 78 (92%) of the 85 assessments. For the remaining seven chemicals, only a single value could be derived (e.g., anatoxin-a, dichloromethane, 2-methylnaphthalene, NDMA, PFOA, PFOS, and triclocarban). 

The retrospective evaluation focused on the comparison of short-term, subchronic, and chronic duration values and was therefore limited to the 63 assessments containing values for all three durations. The duration-specific RfDs and HBG for these 63 assessments are presented in Tables S1 and S2 (see Supplemental Information).

### 3.1. Comparison of Duration-Specific Reference Doses

**Figure 1:**
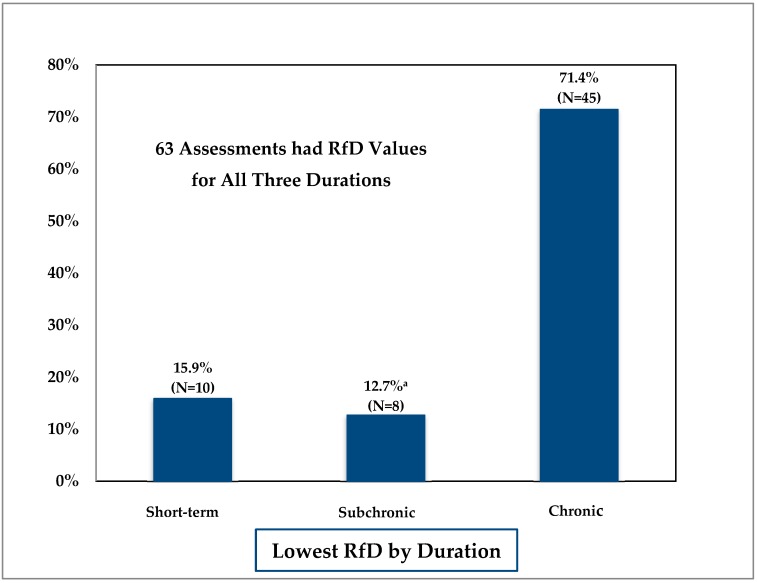
Comparison of reference doses (RfDs) across durations. ^a^ Includes four assessments in which RfD was slightly lower than the chronic RfD due to dose selection.

As expected, based on established toxicological trends, the MDH found that, in general RfDs decreased with increasing duration (short-term > subchronic > chronic), as shown in Figure 1. 

In 45 of the 63 (71.4%) multiple duration assessments, the calculated chronic RfD was equal to or lower than the selected short-term RfD. For 10 of the 63 (15.9%) assessments, the short-term RfD was the lowest RfD. In 6 of the 10 cases, adverse developmental effects were the basis of the short-term RfD. The six chemicals were benzo[*a*]pyrene, boron, butyl benzyl phthalate, dibutyl phthalate, di-2-ethylhexyl phthalate, and ethylene glycol. Two of the remaining four chemicals were cholinesterase inhibitors (chlorpyrifos and chlorpyrifos oxon). The final remaining two were pharmaceuticals (desvenlafaxine and venlafaxine), where the RfD was based on a minimal therapeutic dose level. The chronic studies upon which the chronic RfD was based did not assess the most sensitive life stages or physiological state (e.g., pregnancy). The chronic RfD must be protective of health effects that could result from less than chronic exposures, therefore the final chronic RfD was set to the lower short-term RfD.

In 8 of the 63 (12.7%) multiple duration assessments the subchronic RfD was the lowest calculated RfD value. In one case, trichloroethylene (TCE), the lower subchronic RfD was based on developmental immune effects observed in a subchronic duration study. For three chemicals (17α-ethinylestradiol, mestranol, and thiamethoxam), the RfD was based on male reproductive system effects observed in a subchronic duration study. In these instances, the subchronic RfD was used as the final RfD for the chronic duration as well to ensure that the chronic RfD was protective of subchronic exposures that could occur within a chronic exposure.

For the remaining four chemical assessments (*N*,*N*-diethyl-meta-toluamide (DEET), sulfamethozine/sulfamethoxazole, triclosan, and xylenes) the subchronic RfD was only slightly lower than the chronic RfD and was due to the different dose levels selected for the subchronic and chronic studies. For example, the NOAEL for the subchronic DEET study was lower than the NOAEL for the chronic study. Benchmark dose estimates, which might have corrected for the difference in dose selection, were not available. Another example is triclosan, in which the lowest dose tested for the subchronic study was a LOAEL whereas the point of departure for the chronic study was a NOAEL. Application of a LOAEL-to-NOAEL uncertainty factor, not differences in toxicological sensitivity, produced a lower subchronic RfD. In these four instances, the calculated chronic RfD rather than the slightly lower subchronic RfD was used as the RfD for the chronic duration.

Although the RfDs, in general, decreased with increasing duration (short-term > subchronic > chronic), the shorter duration RfDs were often not much higher than the longer duration RfDs. This is demonstrated by a geometric mean ratio of 3.5 ± 2.8 for comparison of short-term to chronic RfDs and 1.9 ± 2.0 for comparison of subchronic to chronic RfDs when all 63 assessments are considered. The 95th percentile ratios for short-term to chronic and subchronic to chronic were 24.1 and 5.4, respectively, See Table 3. 

The application of duration-based uncertainty factors, as well as study design factors (e.g., dose selection), can directly influence the comparison of chronic to shorter duration RfDs. The MDH used the standard practice of applying a subchronic-to-chronic uncertainty factor if the study used as the basis of the chronic RfD was less than chronic in duration. The maximum MDH subchronic to chronic RfD ratio was 10, reflecting this practice. However, a factor of 10 was not always applied when a less than chronic study was used. The MDH evaluated the entire data set for each chemical to assess the evidence of increasing progression with increasing duration as a basis for an uncertainty factor of 1, 3, or 10. Of the 63 chronic RfDs, 44 (69.8%) were based on subchronic duration studies. Of these, four incorporated a subchronic-to-chronic uncertainty factor of 10; 20 incorporated a factor of 3; 10 incorporated a factor of 1; and 10 were set to short-term RfD values. 

Limiting the comparison of RfDs across durations to the subset of chemical assessments in which the chronic RfD was based on a chronic animal study produced smaller RfD ratios. The geometric mean and 95th percentile short-term to chronic RfD ratio decrease from 3.4 to 2.9 and 24.1 to 17.1, respectively. Likewise, the geometric mean and 95th percentile subchronic to chronic RfD ratio decrease from 1.9 to 1.6 and 5.4 to 4.6 (see Table 3).

Unlike a comparison of RfDs, the direct comparison of potential points of departure (e.g., NOAELs, LOAELs) across durations is not influenced by the application of uncertainty factors. Several investigators have compared potential points of departure across study durations ([16,17,18,19,20,21,22]) reporting geometric mean ratios of similar magnitude to the current analysis of RfD ratios (see Table 3).

### 3.2. Comparison of Duration-Specific Health-Based Guidance (HBG) Values

The short-term, subchronic, and chronic HBG for the 63 chemicals evaluated in this manuscript are presented in Table S2 (see Supplemental Information). By design and intent, the MDH selected a final HBG from the calculated values to ensure that the final chronic HBG value was protective for all life stages and exposure durations. 

**Figure 2:**
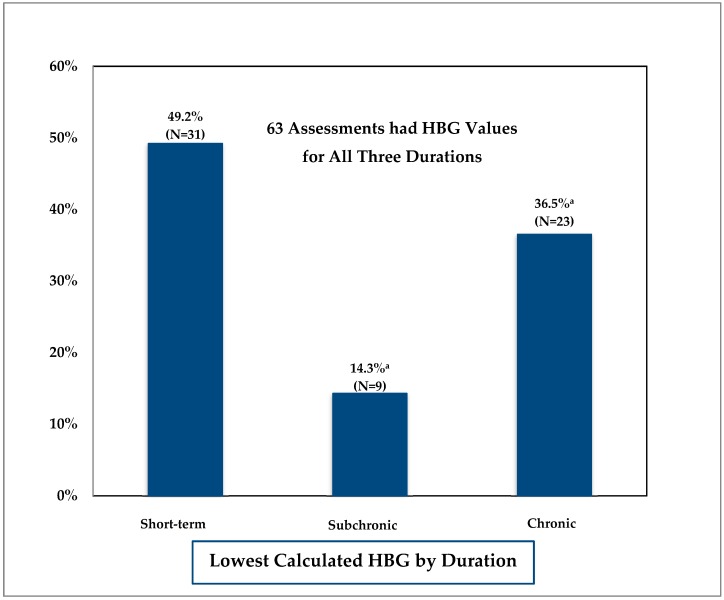
Comparison of health-based guidance (HBG) across durations. ^a^ Includes values that are equal to the lowest value (e.g., calculated subchronic HBG same as short-term HBG or calculated chronic HBG same as short-term or subchronic HBG).

In testing the presumption that HBG would decrease with increasing duration (short-term > subchronic > chronic) the MDH found chronic HBG values to be the lowest (or equal to the lowest shorter duration value) calculated HBG value in only 23 of 63 (36.5%) assessments. See Figure 2. For 31 of the 63 (49.2%) assessments, the short-term HBG was the lowest HBG value. Fifty percent of the short-term to calculated chronic HBG ratios were 0.8 or less and 30 percent of the ratios were 0.5 or less. In the nine remaining assessments, the subchronic HBG was the lowest (or equal to the lowest shorter duration value). The 50th percentile subchronic to calculated chronic HBG ratio was 1.0 and 10 percent of the ratios were 0.5 or less.

High intake rates from early life exposure are a key contributing factor in these findings. As shown in Table 2, short-term duration intake rates can be six to nine times higher than chronic intake rates. The difference between the 95th percentile based short-term (0.285 L/kg per day) and chronic (0.044 L/kg per day) water intake rates used by the MDH is 6.5-fold. When shorter- and longer-duration RfDs are similar in magnitude, the high intake rate from the shorter-duration resulted in a lower shorter-duration HBG value relative to the longer-duration HBG value. Thus, in order to ensure that the longer-duration HBG value, which encompasses all life stages, is protective for higher shorter-term exposure that occurs within its defined time span, the longer-duration HBG value must be set equal to the lower, shorter-duration HBG.

The choice of RSC can also impact the magnitude of each duration-specific HBG. The purpose of the RSC is to account for multiple sources of exposure. As noted in the introduction, the US EPA Office of Water typically does not apply an RSC factor to exposure durations that are less than chronic in duration. However, accounting for both dietary and nondietary (e.g., water ingestion) exposures was among the specific changes recommended by NAS [1].

The narrower range of environments encountered by an infant during the first few month of life justified the use of an RSC of 0.5 for short-term (infant) exposures for chemicals not considered highly volatile. For pharmaceuticals available only through prescription, the MDH used an RSC of 0.8 because exposure from non-water sources, apart from prescription use, is unlikely (see Appendix K of [3] for further explanation). Shorter duration HBG values for non-highly volatile chemicals were lower than the calculated chronic duration HBGs in 19 of 51 (37%) assessments. 

For the derivation of HBG values for highly volatile chemicals, the MDH used a default RSC factor of 0.2 across all durations since the principal route of exposure is expected to be inhalation for all ages. Eight of the 12 (67%) assessments for highly volatile chemicals had a calculated shorter duration (short-term or subchronic) HBG value that was lower than the calculated chronic HBG value (see Supplemental Table S2). Therefore, when RSC is set equal across all durations, it becomes more likely that the short-term duration HBG will be lower than the chronic HBG. 

As mentioned above, the US EPA Office of Water typically does not apply an RSC factor to exposure durations that are less than chronic in duration. If no RSC factor was used to derive shorter duration HBG and a default RSC factor of 0.2 was always used in deriving chronic HBG, 12 of the 63 (19%) chronic HBG values would exceed the short-term HBG values (see Supplemental Table S3). If an RSC of 0.8, US EPA’s the recommended ceiling value [8], is used for all short-term HBG and an RSC of 0.2, the recommended floor value, is used for all chronic HBG the number of chronic HBG values exceeding the short-term value increases to 16 (25%).

## 4. Conclusions

The present study of multiple duration guidance derived by the MDH over the past 10 years clearly demonstrates the importance of incorporating shorter duration toxicity information and exposure parameters when deriving public health protective water guidance. 

Twenty years ago, NAS recommended changes to regulatory practice in order to better characterize risk to infants and children. In response to these recommendations the US EPA issued their policy on evaluating risks to children, which stated that the US EPA will consistently and explicitly consider risks to infants and children as part of risk assessment and standard setting [23]. In 2013, this policy was reaffirmed by the US EPA [24]. The World Health Organization has also recently acknowledged the need to identify the most vulnerable, based on windows of sensitivity or high exposure, and to incorporate this information into risk assessment [25]. While there is consensus regarding the importance of characterizing risks to infants and children, methods to characterize these risk have generally not been widely implemented.

In 2007, the MDH implemented a multiple-duration approach which included consideration of “windows of sensitivity” to toxicants as well as periods of high exposures. As expected, RfDs typically decreased as exposure duration increased. Exceptions were developmental toxicants and fast acting neurological agents (e.g., cholinesterase inhibitors). The difference in magnitude of the RfDs, however, was often relatively small, with the majority of chronic RfDs less than four-fold lower when compared to the corresponding short-term RfD. This observation is consistent with those made by other investigators who have compared the magnitude of more direct measures of toxicity (e.g., NOAELs) across exposure durations. 

Combining the short-term RfD with the high, short-term infant water intake yielded short-term duration guidance that was lower (more protective) than chronic guidance for a majority of chemicals evaluated. The main reason for this phenomenon was the high water intake rate in young infants, which is nearly seven-fold higher than in older children and adults. Derivation of short-term duration guidance may be necessary to ensure protection for all periods of susceptibility in the general population, including periods of high water intake and early-life stages.

As acknowledged by the US EPA Technical Panel [2], derivation of RfDs and RfCs has historically focused on the chronic duration. Short-term and subchronic reference values are typically not derived. Derivation of additional RfD and RfC values is resource intensive, one likely significant factor undercutting the implementation of risk assessments that focus on multiple durations. 

In situations where shorter duration reference values are needed to assess risk, the panel identified the use of the chronic reference value to assess shorter duration exposures as one possible option. Based on the relatively small differential between shorter and chronic duration toxicity values for many chemicals (Table 3), utilization of a chronic RfD value, when shorter duration RfDs are not available, appears to be a reasonable screening level approach to assessing whether shorter duration exposures may be of potential concern. The MDH has incorporated the use of chronic RfDs into derivation of rapid assessments (e.g., screening values) for nearly 160 pesticides [26] and 120 pharmaceuticals [27] in water to assist in identifying situations in which potential risks from short duration exposures warrant closer scrutiny.

The MDH’s HBG work, which incorporates US EPA guidance, underscores the importance of evaluating shorter duration periods of high exposure in order to afford susceptible populations such as infants and children, the same level of protection as adults. This retrospective analysis clearly demonstrates the importance of deriving shorter-duration RfDs, whether based on adult or early-life (developmental) toxicity studies, and characterizing risk for high, short-term exposure durations. 

## Figures and Tables

**Table 1:** Defined study exposure duration [2].

Duration	Definition
Short-term	Repeated exposure for more than 24 h, up to 30 days.
Subchronic	Repeated exposure for more than 30 days, up to approximately 10 percent of a lifetime (approximately 90 days in typical laboratory rodent studies)
Chronic	Repeated exposure for more than approximately 10 percent of a lifespan (more than 90 days, to 2 years in typical laboratory rodent studies)

**Table 2:** Age group water ingestion rates (Tables 3-1 and 3-3, consumers only [14]).

Age Group	Mean (L/kg-Day)	Ratio to All Ages	95th Percentile (L/kg-Day)	Ratio to All Ages
Birth to <1 month	0.137	8.6	0.238	5.4
1 to <3 months	0.119	7.4	0.285	6.5
3 to <6 months	0.080	5.0	0.173	3.9
6 to <12 months	0.053	3.3	0.129	2.9
1 to <2 years	0.027	1.7	0.075	1.7
2 to <3 years	0.026	1.6	0.062	1.4
3 to <6 years	0.021	1.3	0.052	1.2
6 to <11 years	0.017	1.1	0.047	1.1
11 to <16 years	0.012	0.8	0.035	0.8
16 to <18 years	0.010	0.6	0.030	0.7
18 to <21 years	0.011	0.7	0.036	0.8
≥21 years	0.016	1.0	0.042	1.0
All ages (lifetime)	0.016		0.044	
Pregnant women	0.014	0.9	0.043	1.0
Lactating women	0.026	1.6	0.055	1.3

**Table 3:** Descriptive statistical summary analysis comparing measures of oral toxicity across exposure durations.

Study	Comparison Parameter ^a^	Number of Chemicals	Geometric Mean ± GSD	95th Percentile
**Short-term to Chronic**				
Current Analysis	RfD	63	3.5 ± 2.8	24.1
	RfD ^b^	18	2.9 ± 2.4	17.1
Batke et al. [16]	NOAEL	14	3.4 ± 3.7	29.2
Zarn et al. [17]	NOAEL (rat)	107	4.3 ± 4.7	53.2
	NOAEL (mouse)	56	3.4 ± 3.6	23.7
Malkiewicz et al. [18]	NOAEL	26	3.1 ± 2.1	
Groeneveld et al. [19]	NOAEL	35	4.9 ± 3.5	38.6
Kramer et al. [20]	NOAEL	71	4.1 ± 4.4	46
**Subchronic to Chronic**				
Current Analysis	RfD	63	1.9 ± 2.0	5.4
	RfD ^b^	18	1.6 ± 2.0	4.6
Batke et al. [16]	NOAEL	58	1.4 ± 2.1	4.7
Zarn et al. [17]	NOAEL (rat) NOAEL (mouse)	222 99	2.5 ± 3.4 2.2 ± 3.9	17.4 21.4
Malkiewicz et al. [18]	NOAEL	32	2.3 ± 2.0	
Bokkers and Slob [21]	NOAEL benchmark dose	68 189	1.5 ± 5.3 1.7 ± 2.9	22.7 9.9
Groeneveld et al. [19]	NOAEL	70	2.3 ± 3.6	18.4
Pieters et al. [22]	NOAEL	149	1.7 ± 5.6	29

GSD—geometric standard deviation; ^a^ Comparison parameter: no observable adverse effect level (NOAEL); lowest observable adverse effect level (LOAEL); and oral reference dose (RfD); ^b^ Limited to chemical assessments in which comparison is across laboratory animal studies and the chronic RfD is based on a chronic study.

## Supplementary Materials

The following are available online at: http://www.mdpi.com/1660-4601/15/3/512/s1, Table S1: reference dose derivation information for 63 MDH chemical assessments with short-term, subchronic, and chronic values, Table S2: health-based guidance derivation information, Table S3: health-based guidance derivation information—RSC removed from less than chronic values.
